# Kidney Transplantation From Uncontrolled Donation After Circulatory Death Maintained by Normothermic Regional Perfusion: An 8-Year Portuguese Single-Centre Experience

**DOI:** 10.3389/ti.2025.14651

**Published:** 2025-08-26

**Authors:** Ana Pinho, Susana Sampaio, Inês Alencastre, Maria João Polidoro, Margarida Rios, Roberto Roncon-Albuquerque, João Silva, Carlos Silva, Manuel Pestana

**Affiliations:** ^1^ Centro de Referência de Transplante Renal – Adultos, Unidade Local de Saúde São João EPE, Porto, Portugal; ^2^ Faculdade de Medicina da Universidade do Porto, Porto, Portugal; ^3^ Centro de Estatística e Aplicações da Universidade de Lisboa (CEAUL), Lisboa, Portugal; ^4^ Escola Superior de Tecnologia e Gestão do Instituto Politécnico do Porto (ESTG|IPP), Felgueiras, Portugal; ^5^ Gabinete de Coordenação e Colheita de Órgãos para Transplante, Unidade Local de Saúde São João EPE, Porto, Portugal; ^6^ Departamento de Emergência e Medicina Intensiva, Unidade Local de Saúde São João EPE, Porto, Portugal

**Keywords:** kidney transplantation, uncontrolled donation after circulatory death, abdominal normothermic regional perfusion, delayed graft function, primary non-function, brain-death donors, expanded-criteria donors, extracorporeal membrane oxygenation

## Abstract

In January 2016, our hospital started a program of uncontrolled donation after circulatory death (uDCD) to increase organ availability for kidney transplantation. We analysed the results of 523 consecutive kidney transplants (KT) performed from January 2016 to December 2023 in our center and compared the outcomes of 142 KT from uDCD maintained by abdominal normothermic regional perfusion (A-NRP) with those from 194 KT from standard-criteria brain-death donors (SCD) and 187 KT from expanded-criteria brain-death donors (ECD). Primary non-function (PNF) was similar in uDCD (16.9%) and ECD (13.4%, *p =* 0.460) and more common than in SCD (4.6%; *p <* 0.001). In addition, delayed graft function (DGF) differed among the groups, being higher in the uDCD (69.7%), followed by ECD (43.9%) and SCD (37.6%; *p* ≤ 0.05). However, the estimated glomerular filtration rate (eGFR) at 7 years was similar in uDCD and SCD (62.27 ± 18.38 mL/min/1.73 m^2^ vs. 65.48 ± 19.24 mL/min/1.73 m^2^, *p* = 1) and higher than in ECD (47.67 ± 23.05 mL/min/1.73 m^2^, *p* < 0.001). When excluding PNF, the 7-year death-censored graft survival was similar among the three groups (SCD, 91.4%; uDCD, 96.2%; ECD, 82.7%). Despite the increased risk of PNF and DGF, functional and survival outcomes of uDCD KT at 7 years were comparable to those of SCD, thus supporting the use of uDCD kidneys maintained under A-NRP as a successful resource to address organ scarcity.

## Graphical Abstract

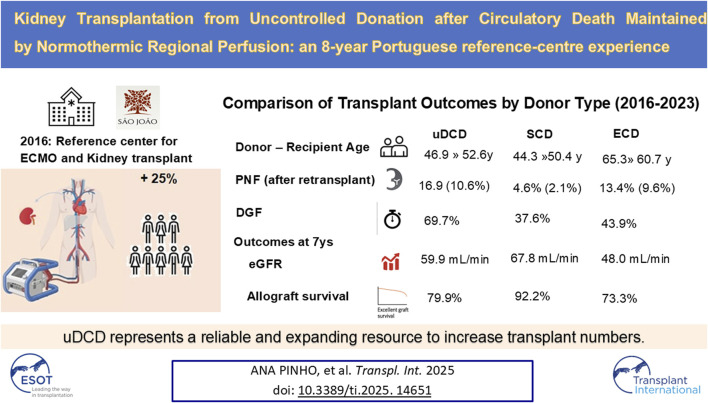

## Introduction

Portugal has one of the highest prevalence rates of chronic kidney disease (CKD) requiring renal replacement therapy in the world (>2000 per million) [1]. Transplantation is the best and most cost-effective therapeutic option for eligible patients with end-stage kidney disease. Most kidney transplants (KTs) are carried out with donors declared dead based on neurologic criteria, that is, donation with brain death donors (DBD). Even considering the upward trend in kidney transplants with DBD observed in the last years, peaking at 35.8 per million in 2023, the time on the active waiting list for transplantation in Portugal is still very long [2].

The necessity to expand the donor pool led to an alteration in the Portuguese law in 2013, which opened up the possibility of organ recovery from uncontrolled donation after circulatory death (uDCD) donors, when the efforts after cardiopulmonary resuscitation are unsuccessful (Maastricht II category) [3].

The growing use of extracorporeal membrane oxygenation (ECMO) in critical care has reinvigorated the interest in uDCD donors. Adapting the ECMO technology to post-mortem A-NRP has been shown to reduce the detrimental effects of warm ischemia leading to better allograft-related outcomes with uDCD donors, compared with the previous technique of *in situ* perfusion [4, 5].

In January 2016 we started a project in Unidade Local de Saúde São João, EPE (ULS São João), the largest tertiary teaching hospital in the North of Portugal serving an urban area of ∼1.5 million inhabitants, to optimize organ donation from uDCD donors after unsuccessful cardiopulmonary resuscitation. For this purpose, an area of integration of pre-hospital emergency with an in-hospital emergency was created, for assistance in refractory out-of-hospital cardiopulmonary arrest, in compliance with the recommendations of the European Council on matters of resuscitation and organ procurement from uDCD donors [6].

In the present study, we report the results of the first 8 years of KTs carried out with uDCD donors in our institution and compare the outcomes with those from KTs from DBD donors, including both standard criteria (SCD) and expanded criteria (ECD).

## Patients and Methods

### Study Design

This retrospective study entails a cohort of all consecutive KTs performed at ULS São João from January 2016 to December 2023 (n = 523). During this period, 142 KTs were carried out with uDCD donors, 194 from SCD and 187 from ECD donors, defined according to the United Network for Organ Sharing (UNOS) [7].

Clinical data were collected using a standardized case report form.

The KTs were followed up for a minimum of 6 months and a maximum of 96 months. The last follow-up date was 31 December 2023, unless graft loss or death occurred first. The follow-up period analysed was 7 years for at least 1 year of follow-up for each patient.

The main outcomes studied were 7-year graft survival (both uncensored and censored by death), recipient survival and longitudinal analysis of graft function over 8 years.

In addition, donor and recipient pre-transplant factors and post-transplant variables were assessed and analysed concerning their relevance in the above-described outcomes. Donor characteristics were (Table 1): age, gender, cause of death (cardiac arrest, stroke, trauma, and other), preoperative serum creatinine (SCreat, mg/dL), warm ischemia time (WIT, min), time on A-NRP (min) and cold ischemia time (CIT, hours). Recipient characteristics were (Table 2): age, gender, etiology of CKD (polycystic kidney disease, glomerulonephritis, diabetes, and others including undetermined), dialysis modality (haemodialysis or peritoneal dialysis), dialysis vintage, previous kidney transplant, immunosuppressive induction therapy and immunological risk based on panel reactive antibody (PRA, %) and human leukocyte antigen (HLA) mismatch levels.

**TABLE 1:** Donor characteristics according to donation group.

	uDCD	SCD	ECD	p value		
	(n = 142)	(n = 194)	(n = 187)	uDCD vs. SCD	uDCD vs. ECD	SCD vs. ECD
Age (years), mean (SD)	46.9 (11.2)	44.3 (11.6)	65.3 (6.4)	0.114	**<0.001**	**<0.001**
Gender, n (%)				0.091	**0.032**	0.571
female	40 (28.2)	74 (38.2)	67 (35.8)	0.071	0.171	0.690
male	102 (71.8)	120 (61.8)	120 (64.2)			
Cause of death, n (%)						
Cardiac arrest	138 (97.2)	54 (27.8)	19 (10.2)	**<0.001**	**<0.001**	0.006
Stroke	4 (2.80)	73 (37.6)	111 (59.4)	**<0.001**	**<0.001**	**<0.001**
Polytraumatism	__	67 (34.6)	57 (30.4)	**<0.001**	**<0.001**	0.44
Preoperative serum creatinine (mg/dL), mean (SD)	1.2 (0.33)	0.88 (0.38)	0.75 (0.22)	**<0.001**	**<0.001**	<0.001
Warm ischemia time (min),mean (SD)	93.8 (32.1)	__	__	__	__	__
A-NRP (min), mean (SD)	177.7 (31.9)	__	__	__	__	__
Cold ischemia time (hours), mean (SD)	14.5 (3.5)	13.1 (5.8)	14.8 (5.8)	0.031	0.860	**0.0150**

Bold p values indicate significant variables.

A-NRP, abdominal normothermic regional perfusion; ECD, expanded‐criteria brain‐dead donors; SCD, standard‐criteria brain‐dead donors; SD, standard deviation; uDCD, uncontrolled donation after circulatory death.

**TABLE 2:** Recipient characteristics according to donation group.

	uDCD	SCD	ECD	p value		
	(n = 142)	(n = 194)	(n = 187)	uDCD vs. SCD	uDCD vs. ECD	SCD vs. ECD
Age (years), mean (SD)	52.6 (11)	50.4 (10.4)	60.7 (7.9)	0.170	**<0.001**	**<0.001**
Gender, n (%)						
female	44 (31.0)	79 (40.7)	64 (34.2)	0.090	0.610	0.230
male	98 (69.0)	115 (59.3)	123 (65.8)			
Etiology of Chronic Kidney Diseased, n (%)						
Glomerulonephritis	29 (20.4)	54 (27.8)	34 (18.2)	0.161	0.661	0.030
Polycystic kidney disease	27 (19.0)	22 (11.3)	31 (16.6)	0.061	0.630	0.194
Diabetes	15 (10.6)	9 (4.6)	38 (20.3)	0.063	0.029	<0.001
HTA	10 (7.0)	14 (7.3)	3 (1.6)	1.000	0.024	0.021
Unknown	37 (26.1)	50 (25.8)	47 (25.1)	1.000	0.890	0.961
Others	24 (16.9)	45 (23.2)	34 (18.2)	0.141	0.663	0.321
Dialysis modalilty, n (%)						
Hemodialysis	115 (81.0)	164 (84.5)	162 (86.7)	0.191	0.461	0.651
Peritoneal Dialysis	26 (18.3)	28 (14.5)	24 (12.8)	0.211	0.401	0.743
Pre-emptive	1 (0.7)	2 (1.0)	1 (0.5)	__	__	__
Dialysis vintage (months), mean (SD)	50.5 (20.5)	64.9 (38.1)	61.2 (32.8)	**<0.001**	**0.001**	0.67
Previous Kidney transplant, n (%)	__	22 (11.3)	14 (7.5)	__	__	0.15
HLA-ABDR mismatches, n (%)						
0–3	69 (48.6)	116 (59.8)	83 (44.4)	0.541	0.930	0.372
4–6	73 (51.4)	78 (40.2)	104 (55.6)			
PRA, n (%)^a^						
0%	122 (85.9)	146 (75.3)	145 (77.5)	**0.010**	**0.030**	0.791
1%–20%	13 (9.2)	31 (15.9)	34 (18.2)	0.056	**0.010**	0.591
>20%	7 (4.9)	17 (8.8)	8 (4.3)	0.230	1.000	0.140
Induction therapy, n (%)						
ATG-based protocols	139 (97.8)	86 (44.3)	63 (33.7)	**<0.001**	**<0.001**	**0.041**
Basiliximab	3 (2.2)	108 (55.7)	124 (66.3)	**<0.001**	**<0.001**	**0.032**

Bold p values indicate significant variables.

^a^ Pre-formed donor-specific antibodies were a criterion to rule out transplantation.

ADPKD, autosomal dominant polycystic kidney disease; ATG, Anti-thymocyte globulin; CMV, Cytomegalovirus; CKD, chronic kidney disease; RRT, Renal replacement therapy; HLA, Human leukocyte antigen; PRA, Panel reactive antibodies; SD, standard deviation.

Post-transplant characteristics included in the analysis were (Table 3): primary non-function (PNF), delayed graft function (DGF), biopsy-proven acute rejection (BPAR), surgical complications, hospitalization days, and months of follow-up.

**TABLE 3:** Post-transplant characteristics according to donation group.

	uDCD	SCD	ECD	p value		
	(n = 142)	(n = 194)	(n = 187)	uDCD vs. SCD	uDCD vs. ECD	SCD vs. ECD
PNF, n (%)	24 (16.9)	9 (4.6)	25 (13.4)	**<0.001**	0.462	**0.005**
Surgical complication	13 (9.2)	5 (2.6)	19 (10.2)			
Rejection	4 (2.82)	1 (0.5)	1 (0.5)			
Others	7 (4.9)	3 (1.6)	5 (2.7)			
PNF after retransplantation, n (%)	15 (10.6%)	4 (2.1%)	18 (9.62%)	**<0.001**	0.371	**0.003**
DGF, n (%)	99 (69.7)	73 (37.6)	82 (43.9)	**<0.001**	**<0.001**	0.050
Sessions of HD until decrease of Cr, median (IQR)	13 (7.5–19)	7 (3–11)	7.5 (3–12)	**<0.001**	**<0.001**	0.813
Biopsy-proven acute rejection, n (%)	18 (12.7)	14 (7.2)	18 (9.6)	0.130	0.481	0.510
Acute cellular rejection	4 (2.8)	5 (2.6)	4 (2.1)			
Acute humoral rejection	2 (1.4)	3 (1.6)	3 (1.6)			
Bordline	12 (8.5)	7 (3.6)	10 (5.35)			
Hospital stay (days), mean (SD)	25.2 (15.2)	15.5 (12.6)	19.8 (16.4)	**<0.001**	**0.008**	**0.013**
*De novo* donor-specific antibodies, n (%)	8 (4.1%)	10 (5.3%)	5 (3.5%)	0.313	0.194	0.740
Follow-up (months), median (IQR)	31.5 (8–64)	38.5 (22–67)	24 (9.5–55)	0.551	0.470	**0.003**

Bold p values indicate significant variables. Cr, Creatinine; DGF, delayed graft function; HD, hemodialysis; IQR, 25%–75% quartil; PNF, primary nonfunction; SD, standard deviation.

The study was performed according to the ethical standards in the Helsinki and Istanbul declarations and was approved by the Local Institutional Review Board and Ethics Committee of ULS São João. Due to the retrospective and non-interventional nature of the investigation, the need for specific informed consent was waived.

Notwithstanding the Portuguese law concerning organ retrieval being an opting-out rule system, the local Organ Procurement Office obtained the agreement from each deceased donor’s next of kin. All recipients were informed at the pre-transplant outpatient clinic about the different types of donors and signed an Informed Consent, according to the donor definition, upon admission for the transplant.

### Variables Definitions

Expanded-criteria brain-death donor was defined as a donor >60 years old or >50 years and with 2 of the 3 comorbidities: hypertension (HTA), death due cerebrovascular event, and SCreat >1.5 mg/dL.

Cold ischemia time was defined as the time from the donor´s aortic clamping during the recovery of the organs until the unclamping of the renal artery during the transplant surgery.

Warm ischemia time was defined for uDCD donors as the time elapsed after cardiac arrest until A-NRP was established.

The A-NRP time encompassed the period from the start of A-NRP until organ retrieval.

The immunological risk was assessed based on three PRA groups (0%; 1%–20%; >20%) and the number of mismatches between donor and recipient of HLA-A, HLA-B, and HLA-DR combined; this variable was dichotomized into two groups (0–3; 4–6).

Primary non-function was defined as permanent graft non-functioning leading to the immediate continuation of dialysis therapy, re-transplant or death. Many PNF cases were associated with early technical complications. Due to frequent overlap between events—such as simultaneous arterial and venous thrombosis, or thrombosis with bleeding or hematoma—a precise attribution of a single causal factor was often not possible.

To address this, we grouped all such intraoperative or early postoperative events under a single category: “surgical complications.” This classification reflects the multifactorial nature of early graft failure and minimizes misclassification bias inherent in retrospective analyses.

Delayed graft function was defined as the need for at least one haemodialysis session during the first week post-transplant, with subsequent recovery of kidney function.

All the acute rejection episodes were biopsy-proven and classified as BPARs. In cases of non-satisfactory function, a percutaneous renal graft biopsy was routinely performed post-transplant during the first 10–14 days and repeated 7–10 days after in case of prolonged DGF.

The permanent return to dialysis or re-transplant defined the death‐censored graft loss. Graft survival encompassed graft loss to dialysis or re-transplant as well as recipient death. Patient survival was determined until death, censored to return to dialysis (considering the deaths during the first 3 months post-transplantation), or until the last date of maximum follow-up, 8 years post-transplantation.

The graft function was assessed by the estimated glomerular filtration rate (eGFR, in mL/min/1.73m2) using the CKD-EPI equation [8]. The eGFR value following transplant was measured at 1 month, 3 months, every 6 months until the end of the third year, and annually thereafter.

### Kidney Transplantation Program: Donation After Circulatory Death

The potential uDCD donors in our center must be ≥18 and ≤60 years old, although this is not an exclusion criterion. In addition, standard criteria for selecting uDCD donors were followed regarding neoplastic, infectious, or potentially transmissible diseases [9].

The timeline and order of events for unsuccessful resuscitation before identifying potential uDCD donors were carried out according to the ULS São João protocol [6]. Patients with out-of-hospital cardiac arrest are transported to the emergency department of our hospital on ventilation support and continuous mechanical chest compressions (LUCAS, Physio-Control Inc., Sweden). In case of unsuccessfully advanced life support without inclusion criteria for E-CPR, the intensivist of the emergency department declares death after 10 min (min) of no-touch when no signs of circulation or electrical activity are found. After this, mechanical chest compressions are restarted, and percutaneous cannulation of a femoral artery and vein is performed. An occlusion balloon is placed on the thoracic aorta through the contralateral femoral artery to exclude brain circulation from the extracorporeal circuit. After the establishment of the extracorporeal circuit, chest compressions is terminated.

The nRP is performed using a Maquet CardioHelp system (Maquet, Germany). This technique restores blood flow and oxygenation to the abdominal organs and allows the recovery of kidneys after a prolonged period of WIT. The target pump flow for nRP is 1.75–2.5 L/min, and a constant temperature of 37°C is maintained. Blood samples are obtained every 30 min for blood gases, and biochemistry analysis including serum lactate levels and hematocrit. The maximum preservation time of nRP is 240 min, but it can be extended to 360 min if blood gases, biochemistry, and hematocrit parameters are adequate for organ recovery.

The extraction of organs is performed through a median laparotomy, maintaining nRP support during surgery. Thereafter the kidneys are included in a preservation fluid (Custodiol HTK, Franz Kohler, Germany) and placed in “static” cold storage at 4°C. Implantation surgery is performed within the next 18 h.

In our center, the characteristics of the recipients considered for receiving a kidney from uDCD or DBD donors are similar. However, we avoid performing transplants with uDCD donors in candidates i) waiting for a retransplant, ii) with high immunological risk, iii) with heart failure with depressed ejection fraction and iv) in those undergoing anticoagulation therapy.

All uDCD transplanted patients and DBD recipients with high immunologic risk received rabbit anti-thymocyte globulin (ATG-Fresenius, Fresenius Biotech GmbH, Germany) as induction immunosuppression, at a maximum dose of 2.5 mg/kg/day, with a cumulative target dose of 12.5 mg/kg. High immunologic risk was defined in cases of cPRA >20%, as well as in recipients with prior transplants, the presence of donor-specific antibodies (even if weakly positive), and those with high mismatch levels (HLA 4–6 mismatches). This broader immunologic risk stratification justified the use of ATG in 44.3% of SCD and 33.7% of ECD recipients. In the remaining DBD recipients, we used anti‐CD25 monoclonal antibodies (basiliximab) as induction therapy.

All uDCD and DBD recipients received maintenance immunosuppression with steroids, tacrolimus and mycophenolate mofetil. We start with a 500 mg dose of iv methylprednisolone before and immediately after surgery and transition to 1 mg/kg/day of oral prednisolone on the first-day post-transplantation, with subsequent tapering. Mycophenolate mofetil is started orally on the first day post-transplant at a 500 mg dose twice a day. After the end of the induction therapy, this dose is increased to 750 mg twice a day. Tacrolimus is started at a 0.2 mg/kg/day dose, 2 days before the predicted last dose of anti-thymocyte globulin, to ensure adequate levels by the end of the induction therapy. All patients receive chemoprophylaxis with cotrimoxazole and nistatin. Valganciclovir is administered during the first 6 months after transplant in recipients that receive induction therapy with ATG or in cases of CMV donor (+)/receptor (−) serostatus.

### Statistical Analysis

Categorical variables were expressed as absolute (n) and relative (%) frequencies. Continuous variables were expressed as mean ± standard deviation (SD) or median with interquartile range (IQR: first and third quartiles) if the hypothesis of normality of the distribution was not verified. The hypothesis of normal distribution was tested using the Shapiro-Wilk test.

Categorical variables were compared between groups using Pearson’s chi-square test or Fisher’s exact test when the first cannot be used. Continuous variables were compared using Student´s t-test when data were normally distributed or using the Wilcoxon-Mann-Whitney´s non-parametric test otherwise. The Bonferroni correction was the method used to counteract the multiple comparison problems.

Survival curves for graft and patient survival were obtained using the Kaplan-Meier method, and differences between the groups were compared using the Log-rank test.

PNF and DGF risk factors were analysed using a binary logistic regression model. Relative risks are reported as odds ratios with 95% confidence intervals. Multivariable models included all significant factors in the univariable models and were determined with a forward stepwise procedure.

A p-value <0.05 was considered significant in all tests. The statistical analysis was undertaken using IBM SPSS Statics software (version 26.0) and R software (version 4.2.2).

## Results

**FIGURE 1:**
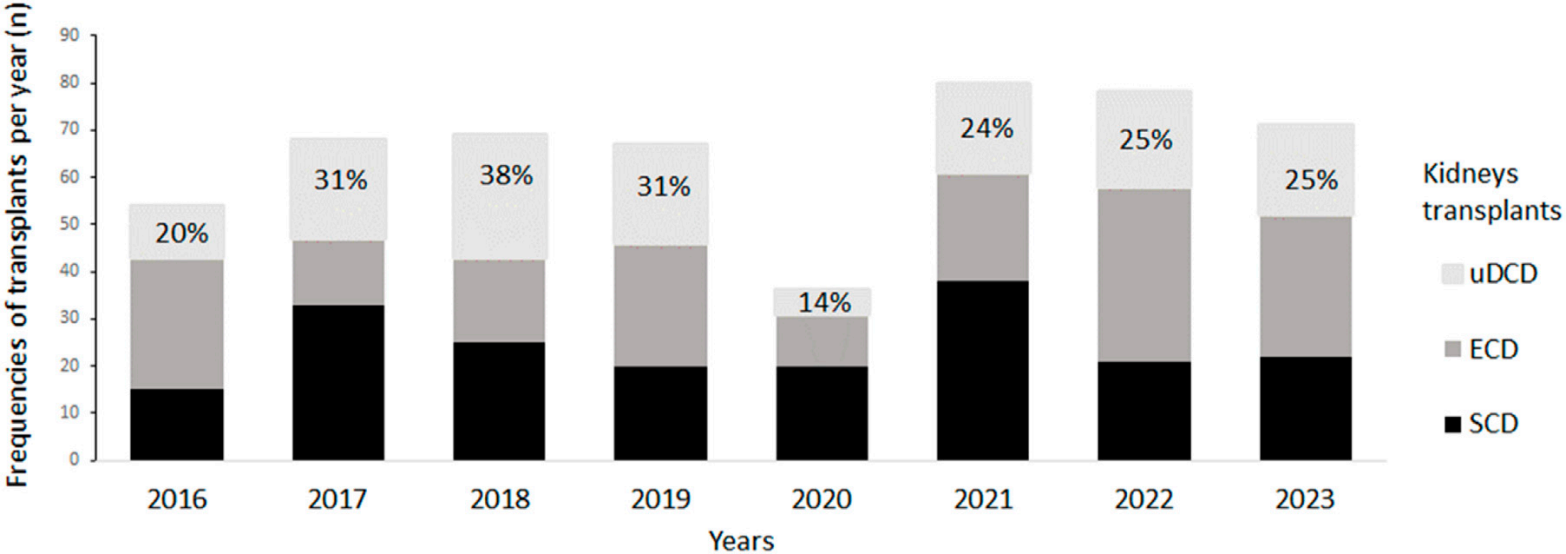
uDCD Kidney transplant impact on the Transplantation Program. ECD, expanded‐criteria brain‐dead donors; SCD, standard‐criteria brain‐dead donors; uDCD, uncontrolled donation after circulatory death.

From January 2016 to December 2023, 523 KT have been carried out in our center: 142 KT from uDCD donors (27.2%), 194 KT from SCD donors (37.1%) and 187 KT from ECD donors (35.7%) (Table 1). The impact of renal transplants from uDCD donors on our program is shown in Figure 1. As can be observed, KTs carried out with uDCD donors represented 20%–38% of the total number of transplants performed each year throughout this period except in 2020 (14%), which corresponded to the first year of the SARS-cov-2 pandemic when the retrieval of organs from uDCD donors was temporarily suspended according to a directive from the Portuguese National Coordination for Transplant.

### Baseline Evaluation

Tables 1 and 2 show an overview of the donor and recipient characteristics according to donor group. The mean age of both donors and recipients in the ECD group was significantly higher (65.3 ± 6.4 and 60.7 ± 7.9 years, respectively) than in the other two groups. However, no significant difference was observed between uDCD (46.9 ± 11.2 and 52.6 ± 11.0 years) and SCD (44.3 ± 11.6 and 50.4 ± 10.4 years).groups regarding the median age of both donors and recipients. Male donors predominated across all groups (uDCD: 71.8%, SCD: 61.8%, ECD: 64.2%), as did male recipients (uDCD: 69.0%, SCD: 59.3%, ECD: 65.8%).

As can be observed in Table 1, the donor cause of death in uDCD group was mainly due to cardiac arrest (97.2%), whereas the donor cause of death in ECD group was mainly due to stroke (59.4%). Preoperative serum creatinine (SCreat) was significantly higher in uDCD donors (1.2 ± 0.33 mg/dL) compared to both SCD (0.88 ± 0.33 mg/dL) and ECD (0.75 ± 0.22 mg/dL) groups. Cold ischemia time (CIT) was significantly lower in the SCD group (13.1 ± 5.8 h) compared to both ECD (14.8 ± 5.8 h) and uDCD (14.5 ± 3.5 h) groups. Kidneys from uDCD donors had a mean warm ischemia time (WIT) of 93.8 min and a mean abdominal normothermic regional perfusion (A-NRP) duration of 177.7 min.

Recipient characteristics of the three groups are presented in Table 2. Recipients from ECD group had an increased percentage of diabetic aetiology (20.3%) in comparison with the other two groups. On the other hand, the uDCD group’s dialysis vintage (50.5 ± 20.5 months) was lower than the other two groups. As expected, PRA in uDCD group (85.9% had 0%) was lower than in the other two groups. So, according to the protocol, the recipients from uDCD group received more commonly ATG (97.8%) as induction therapy in comparison with the other two groups. No differences were observed among recipients from the three groups concerning previous kidney transplants, dialysis modality, and HLA mismatches.

### Clinical Course

Table 3 summarizes the post-transplant outcomes across the three donor groups. PNF occurred significantly less frequently in the SCD group (4.6%) compared to the uDCD (16.9%) and ECD (13.4%) groups. No significant difference in PNF rates was observed between uDCD and ECD. Surgical complications were the leading cause of PNF in all groups, particularly in uDCD (9.2%) and ECD (10.2%).

Regarding patient outcomes following PNF, in the SCD group, 5 out of 9 patients (55.6%) were relisted and all underwent re-transplantation. In the ECD group, 15 of 25 patients (60.0%) were relisted, and 7 (28.0%) received a second transplant. Among the 24 uDCD recipients with PNF, 19 (79.1%) were relisted, and 9 (37.5%) underwent re-transplantation during the study period. Among the 19 patients who were not relisted—either due to medical contraindications or personal decision—all were older than 60 years, with 11 (57.9%) being over 65. Finally, the overall rate of PNF following re-transplantation was 10.6% in the uDCD group (15 cases), 2.1% in the SCD group (4 cases), and 9.6% in the ECD group (18 cases).

As expected, DGF in recipients from the uDCD group (69.7%) was significantly higher than in the other two groups. In addition, DGF in the ECD group (43.9%) was non-significantly higher than that of the SCD group (37.2%). Accordingly, uDCD recipients had a higher median number of HD sessions and days of hospital stay in comparison with the other two groups. On the other hand, no significant difference was observed in the number of BPARs among the three groups.

As can be observed in Table 4, in univariable analysis, both preoperative serum creatinine (OR 5.042; p = 0.048) and WIT >60 min (OR 1.021; p = 0.016) were significant risk factors for PNF in the uDCD group. In contrast, in the BDD group (SCD + ECD), donor age (OR 1.055; p = 0.001) and CIT (OR 1.082; p = 0.021) were significantly associated with PNF. In multivariable analysis, WIT remained a risk factor for PNF in the uDCD group (OR 1.018; p = 0.047), whereas donor age remained significant in the BDD group (OR 1.056; p = 0.002). Additionally, CIT was a significant risk factor for DGF in both uDCD (OR 1.194; p < 0.001) and BDD (OR 1.091; p < 0.001) groups (Table 4).

**TABLE 4:** Risk factors for PNF and DGF (logistic regression) according to donation group.

A. Risk factor for uncontrolled Donor after Circulatory Death (n = 142)						
Univariate logistic analysis	PNF			DGF		
	Odds Ratio	95% CI	p value	Odds Ratio	95% CI	p value
Donor age	1.025	0.983–1.075	0.272	0.993	0.953–1.031	0.739
Preoperative Cr, mg/dL	5.042	1.082–27.46	**0.048**	1.059	0.268–4.087	0.933
Recipient Age	1.003	0.964–1.045	0.876	0.988	0.949–1.028	0.580
Recipient Age >=50	0.863	0.356–2.156	0.746	1.222	0.494–2.938	0.655
Original nephropaty, DM + HTA	0.623	0.138–2.018	0.475	1.891	0.578–8.554	0.338
Dialysis vintage. mo	1.011	0.989–1.031	0.355	1.009	0.988–1.034	0.392
Warm ischemia time (only for uDCD)	1.021	1.004–1.039	**0.016**	1.009	0.991–1.028	0.334
Cold ischemia time, h	1.009	0.886–1.148	0.884	1.194	1.047–1.379	**<0.001**
Multivariate logistic analysis	Odds Ratio	95% CI	p value	Odds Ratio	95% CI	p value
Preoperative Cr, mg/dL	4.796	0.797–36.982	0.104	NA		
Warm ischemia time	1.018	0.096–1.987	**0.047**	NA		

B. Risk factor for Brain Donor Death groups (n = 381)						
Univariate logistic analysis	PNF			DGF		
	Odds Ratio	95% CI	p value	Odds Ratio	95% CI	p value
Donor age	1.055	1.022–1.092	**0.001**	1.020	1.003–1.036	**0.017**
Polytraumatism cause of death	0.723	0.310–1.547	0.424	0.757	0.478–1.189	0.230
Preoperative Cr, mg/dL	0.348	0.059–1.512	0.201	1.606	0.787–3.319	0.194
Recipient Age	1.036	0.999–1.078	0.067	1.000	0.981–1.021	0.898
Recipient Age >=50	1.684	0.719–4.617	0.264	0.901	0.561–1.464	0.683
Original nephropaty, DM + HTA	0.842	0.227–2.092	0.733	1.192	0.677–2.091	0.539
Dialysis vintage, mo	0.999	0.988–1.008	0.948	1.002	0.996–1.007	0.593
Cold ischemia time, h	1.082	1.013–1.161	**0.021**	1.095	1.054–1.140	**<0.001**
ATG-based protocols	0.942	0.329–2.192	0.827	1.172	0.677–2.121	0.639
PRA (%)	1.006	0.976–1.028	0.595	0.991	0.972–1.007	0.313
Multivariate logistic analysis	Odds Ratio	95% CI	p value	Odds Ratio	95% CI	p value
Donor age	1.056	1.018–3.044	**0.002**	1.013	0.997–1.031	0.091
Cold ischemia time, h	1.065	0.037–1.799	0.072	1.091	1.050–1.136	**<0.001**

Bold p values indicate significant variables. ATG, Anti-thymocyte globulin; DGF, delayed graft function; HD, PNF, primary nonfunction; NA, not applicable.

**FIGURE 2:**
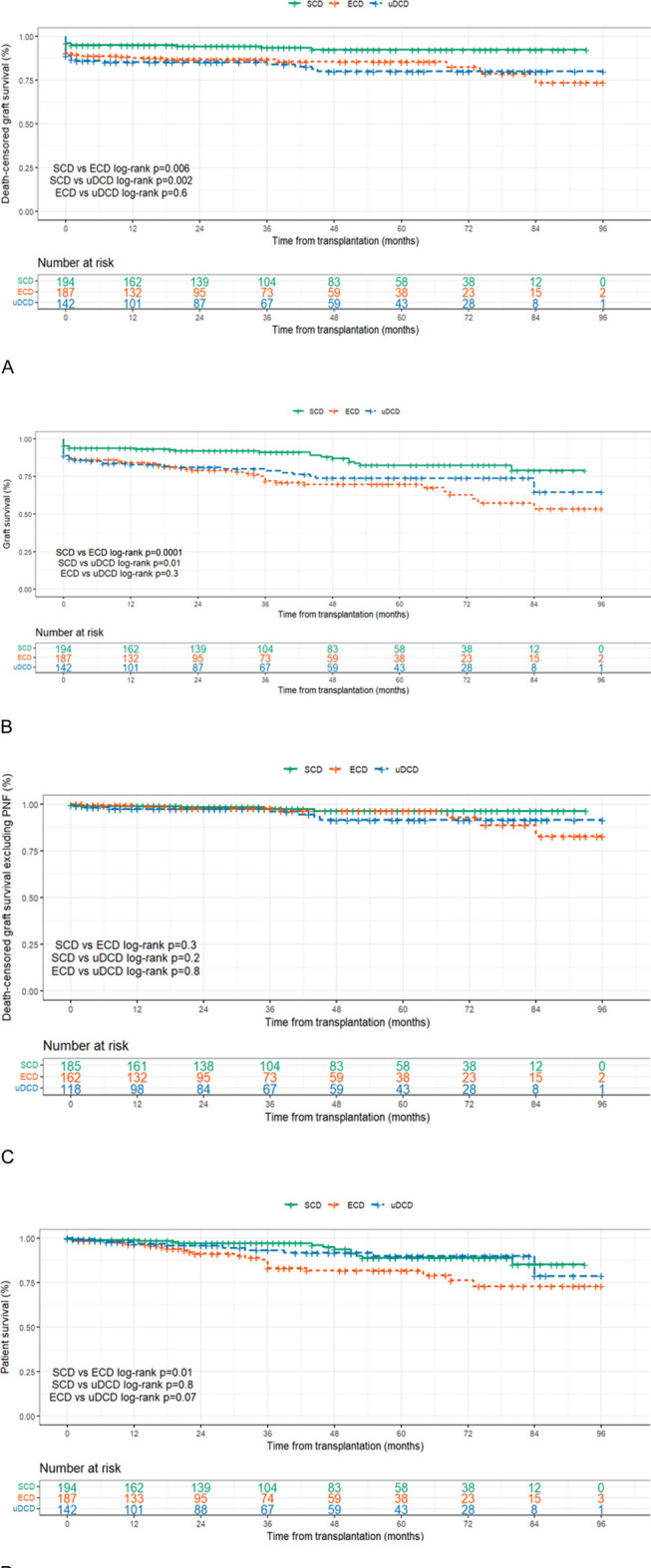
**(A)** Death-censored graft survival. **(B)** Graft survival (death not censored). **(C)** Death-censored graft survival excluding PNF. **(D)** Patient survival. ECD, expanded‐criteria brain‐dead donors; SCD, standard‐criteria brain‐dead donors; uDCD, uncontrolled donation after circulatory death.

The graft survival rate, both death-censored and uncensored, was significantly higher in the SCD group than in the ECD group (Table 5; Figures 2A,B). Death-censored graft survival at 7 years was 92.2% in the SCD group, 79.9% in the uDCD group, and 73.3% in the ECD group (p = 0.003 for SCD vs. ECD; p = 0.082 for SCD vs. uDCD). Similarly, overall graft survival not censored for death was also superior in the SCD group (78.9%) compared to uDCD (64.6%) and ECD (53.5%), with statistically significant differences (p < 0.001 for SCD vs. ECD; p = 0.010 for SCD vs. uDCD).

**TABLE 5:** Long outcomes according to donation group.

	uDCD	SCD	ECD	p value			p value
				uDCD vs. SCD	uDCD vs. ECD	SCD vs. ECD	
No. of patients	**142**	**194**	**187**				
eGFR (ml/min/1.73m2), mean (SD) {number}							
1- year	60.66 (21.55) {113}	69.37 (23.74) {173}	50.28 (19.08) {155}	**0.002**	**<0.001**	**<0.001**	**<0.001**
5- year	59.01 (23.47) {58}	67.07 (25.63) {77}	53.01 (22.61) {56}	0.136	0.381	0.003	
7- year	59.90 (23.69) {26}	67.80 (22.74) {34}	47.99 (23.52) {25}	0.396	0.167	0.005	
Death-censored graft survival, % (SE) {number at risk}							
1- year	85.1 (0.03) {101}	94.8 (0.016) {162}	87.4 (0.025) {132}	0.082	0.283	**0.003**	0.014
5- year	79.9 (0.038) {43}	92.2 (0.022) {58}	85.4 (0.028) {38}				
7- year	79.9 (0.038) {8}	92.2 (0.022) {12}	73.3 (0.071) {15}				
Graft survival (death not censored),% (SE) {number at risk}							
1- year	82.8 (0.032) {101}	93.8 (0.017) {162}	84.2 (0.027) {132}	**0.010**	0.311	**<0.001**	0.007
5- year	73.9 (0.042) {43}	82.4 (0.035) {58}	69.7 (0.040) {38}				
7- year	64.6 (0.094) {8}	78.9 (0.048) {12}	53.5 (0.068) {15}				
Death-censored graft survival excluding PNF, % (SE) {number at risk}							
1- year	97.4 (0.015) {98}	98–9 (0.008) {161}	98.6 (0.010) {132}	0.200	0.810	0.310	0.121
5- year	91.4 (0.032) {43}	96.2 (0.017) {58}	96.3 (0.019) {38}				
7- year	91.4 (0.032) {8}	96.2 (0.017) {12}	82.7 (0.077) {15}				
Patient survival, % (SE) {number at risk}							
1- year	96.7 (0.016) {101}	99.0 (0.007) {162}	96.3 (0.0149) {133}	0.801	0.070	**0.010**	0.309
5- year	89.9 (0.034) {43}	88.9 (0.032) {58}	81.6 (0.039) {38}				
7- year	78.6 (0.109) {8}	85.1 (0.048) {12}	72.8 (0.0596) {15}				

Bold p values indicate significant variables.

uDCD, uncontrolled donation after circulatory death; SCD, standard‐criteria brain‐dead donors; ECD, expanded‐criteria brain‐dead donors; SD, standard deviation; SE, standard error.

However, when excluding cases of primary non-function (PNF), the death-censored graft survival improved notably in all groups, reaching 91.4% in uDCD, 96.2% in SCD, and 82.7% in ECD. Under these conditions, no significant difference in death-censored graft survival was observed among the three groups (p = 0.121), reinforcing the impact of early graft loss on long-term outcomes (Table 5; Figure 2C).

**FIGURE 3:**
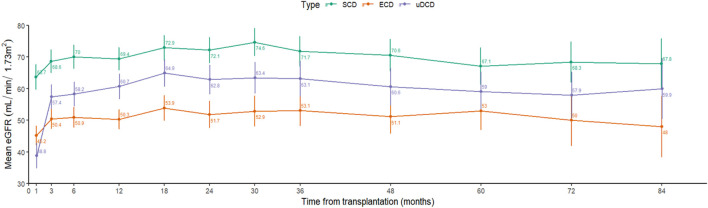
Longitudinally eGFR according to donor type. eGFR, estimating glomerular filtration, using the CKD-EPI equation; ECD, expanded‐criteria brain‐dead donors; SCD, standard‐criteria brain‐dead donors; uDCD, uncontrolled donation after circulatory death.

The longitudinal analysis of allograft kidney function in the three groups is shown in Figure 3 and Table 5. As observed, mean eGFR was consistently higher in the SCD group (69.37 mL/min/1.73 m^2^ at 12 months) than in the ECD group (50.28 mL/min/1.73 m^2^). In the uDCD group, eGFR increased from 1 to 3 months and stabilized around 60.66 mL/min/1.73 m^2^ at 1 year, remaining stable thereafter. This resulted in a significantly higher eGFR in the uDCD group compared to the ECD group at 1 year and beyond. No significant difference was observed in eGFR between SCD (67.80 mL/min/1.73 m^2^) and uDCD (59.90 mL/min/1.73 m^2^; p = 0.396)groups over the 7-year follow-up (Table 5; Figure 3)univariablemultivariablemultivariable

## Discussion

The present study examined the results of the first 8 years since the beginning of our uDCD program and compared the outcomes with those of KTs from BDD, including both SCD and ECD.

Our uDCD allograft function and survival results agree well with other published series in the literature, mainly of uDCD KTs in Europe [10–15]. In addition, the longitudinal analysis of our uDCD KTs showed that both the functional and the survival outcomes were comparable to those observed with KTs from BDD namely SCD. This increased the kidney donor pool in our institution by 14%–38%, thus representing an excellent additional source of organs for transplantation.

We found that PNF was a very relevant cause of KT failure in both uDCD and ECD groups, occurring in 16.9% and 13.4% of patients, respectively. Surgical complications were the most relevant cause in all donor types, but especially in uDCD and ECD. In fact, our previous experience published by Manso et al. [16] had already identified vascular complications—such as arterial or venous thrombosis—as the leading cause of PNF in uDCD recipients, particularly when combined with prolonged ischemia. These findings are consistent with the surgical etiology observed in the current cohort.

The elevated rate of PNF in the ECD group (13.4%) may be explained by the intrinsic characteristics of expanded criteria donors, namely advanced donor age and comorbidities such as hypertension and cerebrovascular disease. These factors are known to negatively affect graft viability. In our cohort, the ECD group also presented with significantly higher pre-retrieval serum creatinine and prolonged cold ischemia times, both associated with poor early graft outcomes.

Although the underlying causes of PNF should be further investigated, the incidence of PNF in the uDCD group was within the range reported by other studies (14.7%–19.6% [10]), including both controlled and uncontrolled DCDs. In adjusted analysis, serum creatinine and a WIT >60 min were the only parameters significantly associated with PNF in uDCD KTs. Of note, no PNF episodes were observed in the uDCD group during 2020, which coincided with a lower mean WIT of 54 min, further supporting the implementation of a WIT-based allocation policy to reduce the risk of PNF in this population.

Abdominal normothermic regional perfusion (A-NRP) is an organ preservation strategy consisting in a modified bypass extracorporeal circulation that re-establishes the flow of oxygenated blood to abdominal organs after cardiac arrest. This technique is being increasingly used to minimize the warm ischemic injury in uDCD, thus contributing to improved organ transplantation outcomes, including PNF, in comparison with kidneys preserved in static cold settings [17]. Since the start of our program, we have always used A-NRP in all KTs from uDCD. Therefore, this strategy cannot be used in the future to decrease the PNF rate. The absence of post-mortem machine perfusion, particularly in uDCD and ECD donors, which differs from practices in other transplant programs and could affect early graft outcomes such as PNF and we consider in future their use.

We found that our uDCD KTs showed a high rate of DGF (69.2%), as reported by other series (80.9% [12], 76% [14], and 73.7% [10]). However, the DGF rates in the uDCD group had no impact on either short- or long-term graft function and survival. This evidence, although previously reported [17–20] is not consistent in other studies [21, 22]. As we systematically used anti-thymocyte globulin induction in uDCD KTs with low immunologic risk, we propose that the lower rate of DGF observed in our uDCD population compared with other series may be explained by the immunosuppression regimen. Additionally, our immunosuppression regimen may also contribute to the reduced incidence of BPAR episodes observed in the uDCD group (12.7%).

Evidence has been gathered favoring the benefit of *ex-vivo* machine perfusion techniques to improve quality and recondition organs for transplant, minimizing graft ischemia-reperfusion injury and reducing of immunogenicity while allowing the real-time monitoring of the harvested organ function, including the measurement of physiological and molecular markers, particularly in the DCD scenario where WIT may impair importantly the quality of the retrieved organs. Although criteria for kidney machine perfusion to improve graft survival/function are still pending this may come to be a useful resource to decrease both PNF and DGF in DCD KTs.

Kidney function from our uDCD KTs at 7 years (59.9 ± 23.69 mL/min/1.73 m^2^) was comparable to other series dealing with uDCD programs [23]. In addition, the longitudinal analysis showed that from 3 months onwards, the eGFR of uDCD KTs was maintained at intermediate values to that observed in the other two groups and, at 7 years, the eGFR of uDCD KTs did not differ significantly from that observed in either SCD or ECD group. This occurred in a scenario where the renal function in the SCD group was maintained significantly higher than that of the ECD group.

Our findings are also in line with those previously reported by a French group [24], which demonstrated that the functional outcomes—particularly overall graft survival—of kidneys transplanted from uncontrolled DCD donors were comparable to those of ECD grafts. This reinforces the notion that, when properly preserved using strategies such as A-NRP, uDCD kidneys can achieve acceptable long-term performance. Historically, in our center, uDCD kidneys have been preferentially allocated to older recipients, mirroring the policy for ECD grafts. However, based on both the literature and the outcomes from our cohort, we are now considering broadening the recipient selection criteria. While our results do not support strict age restrictions, careful assessment of individual recipient risk remains essential. These findings are already influencing our allocation policy and may help optimize graft utility in future uDCD programs.

Moreover, the graft survival when censored to death was not different among KTs from the three groups. The 7-year death-censored graft survival rate of uDCD KTs in our study (79.9% and 91.4%, when excluding PNF) is very motivating and comparable to the previously cited reports [14, 15, 23, 25]. In the same way, patients’ survival rates after 7 years were similar among KTs from the three types of donors.

In addition to the classic statistical analysis aiming at the comparative evaluation of the data collected in this study, we have used a linear and multivariable mixed regression model associated with graft function trajectories, where patients may drop out during the study period because of the initiation of renal replacement therapy. To our knowledge, this is the best approach to analyze graft function data longitudinally over a long follow-up period, with application of robust statistical analyses (both univariable and multivariable) to adjust for relevant confounders.

Implementing uDCD transplantation programs is highly demanding and puts significant pressure on hospital teams. Still, an increase in the number of studies from different centers across the globe may have a transforming impact on the current panorama and lead to the better use of uDCD organs for the benefit of kidney patients [18, 25, 26].

Our study has limitations stemming from the retrospective analysis in a single center despite data collected prospectively according to the unit’s data management system. The retrospective and single-center nature of the analysis, which may limit generalizability, but the size of the uDCD cohort, which is one of the largest reported to date with long-term follow-up.

However, this study innovates by comparing the outcomes of uDCD KTs with those from BDD, including both SCD and ECD groups, over a 7-year follow-up period.

In conclusion, our study reports the results of the first program of uDCD KTs in Portugal and compares the outcomes of uDCD KTs vs. BDD KTs, including both SCD and ECD KTs. Overall, our results show that uDCD KTs have a long-term performance that is very similar to BDD KTs concerning renal function as well as graft and patient survival, thus representing a valuable source of organs that should be considered for the benefit of patients.

## Data Availability

Anonymized data supporting this study’s findings are available from the corresponding author upon reasonable request, in compliance with institutional privacy and ethical regulations.
